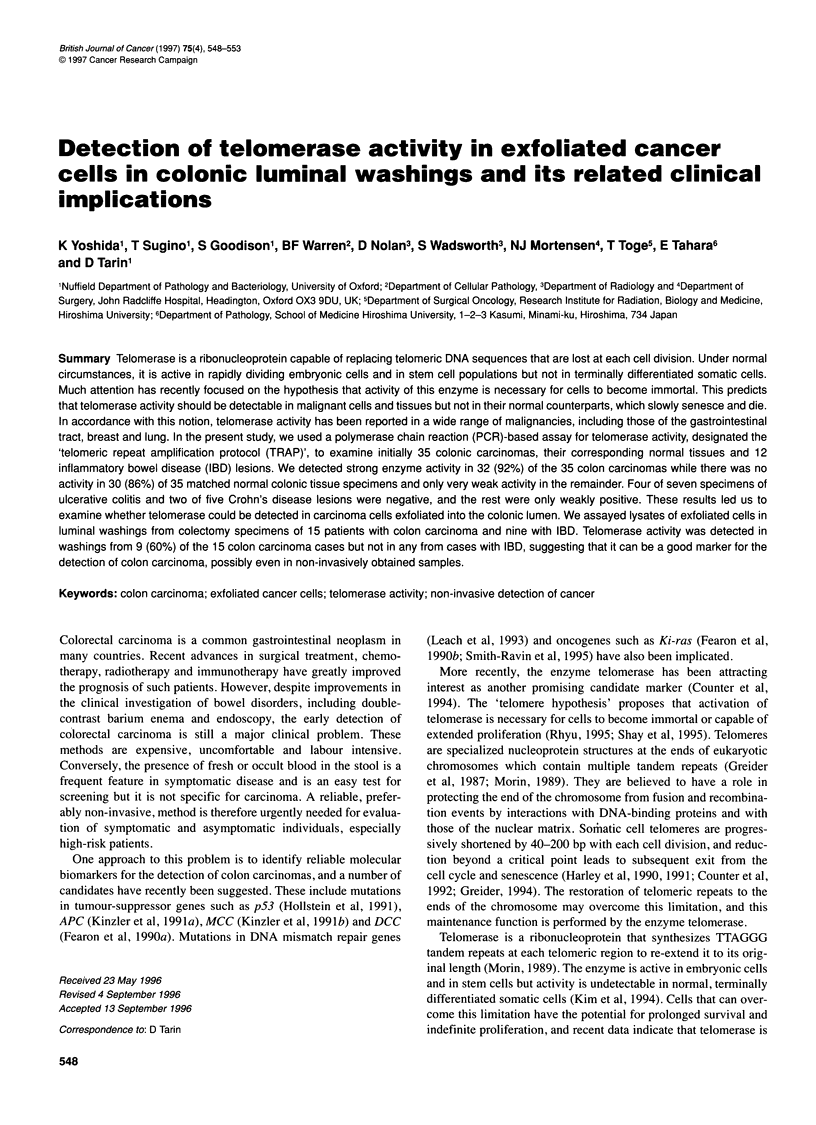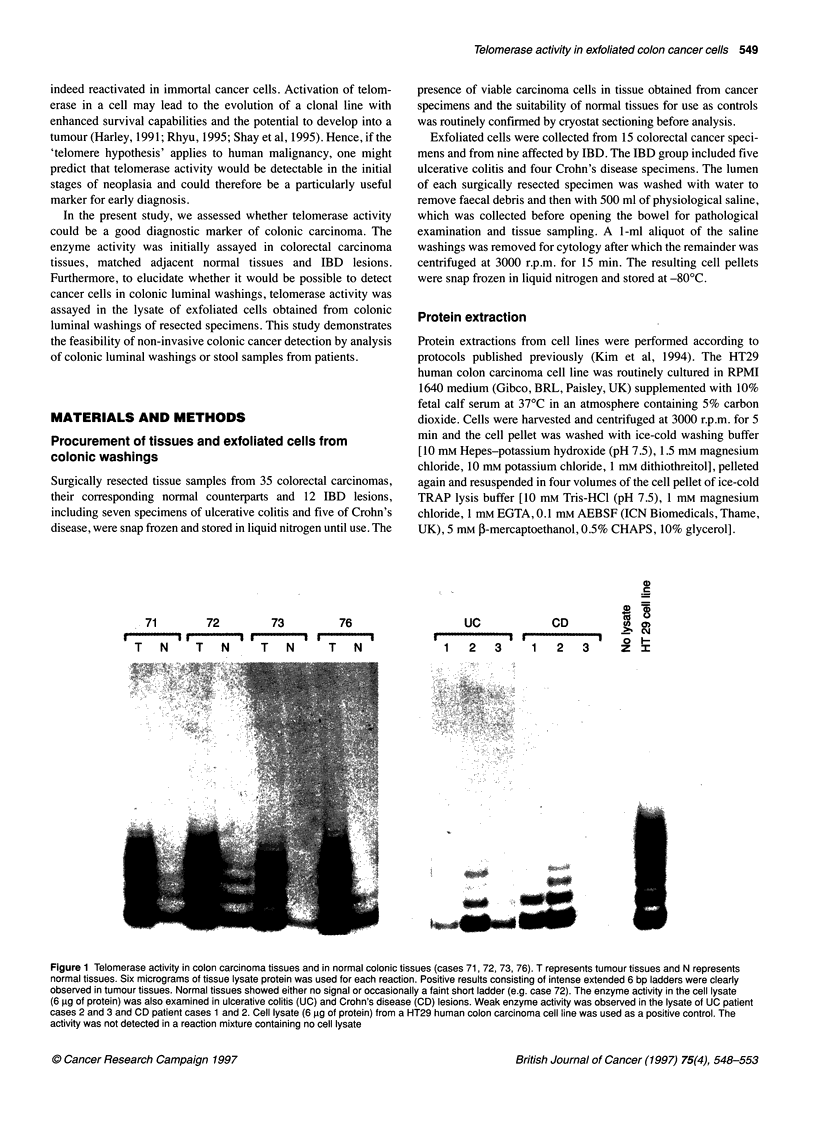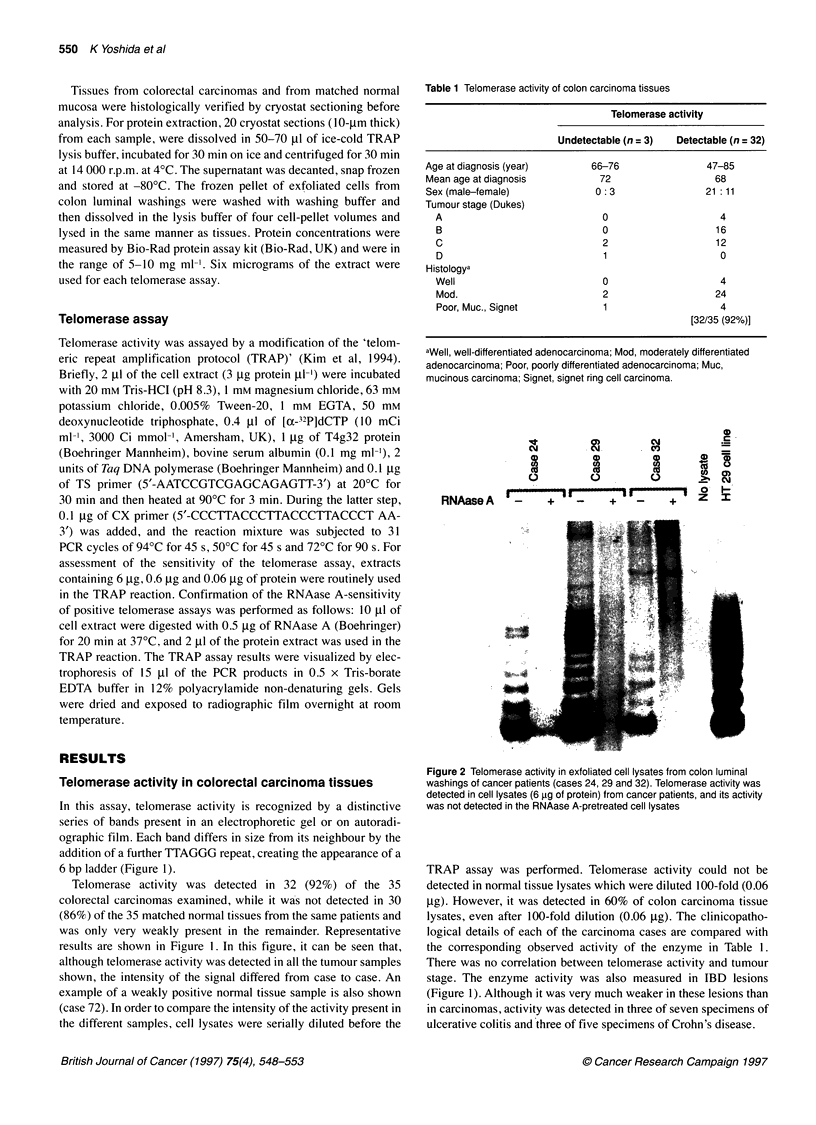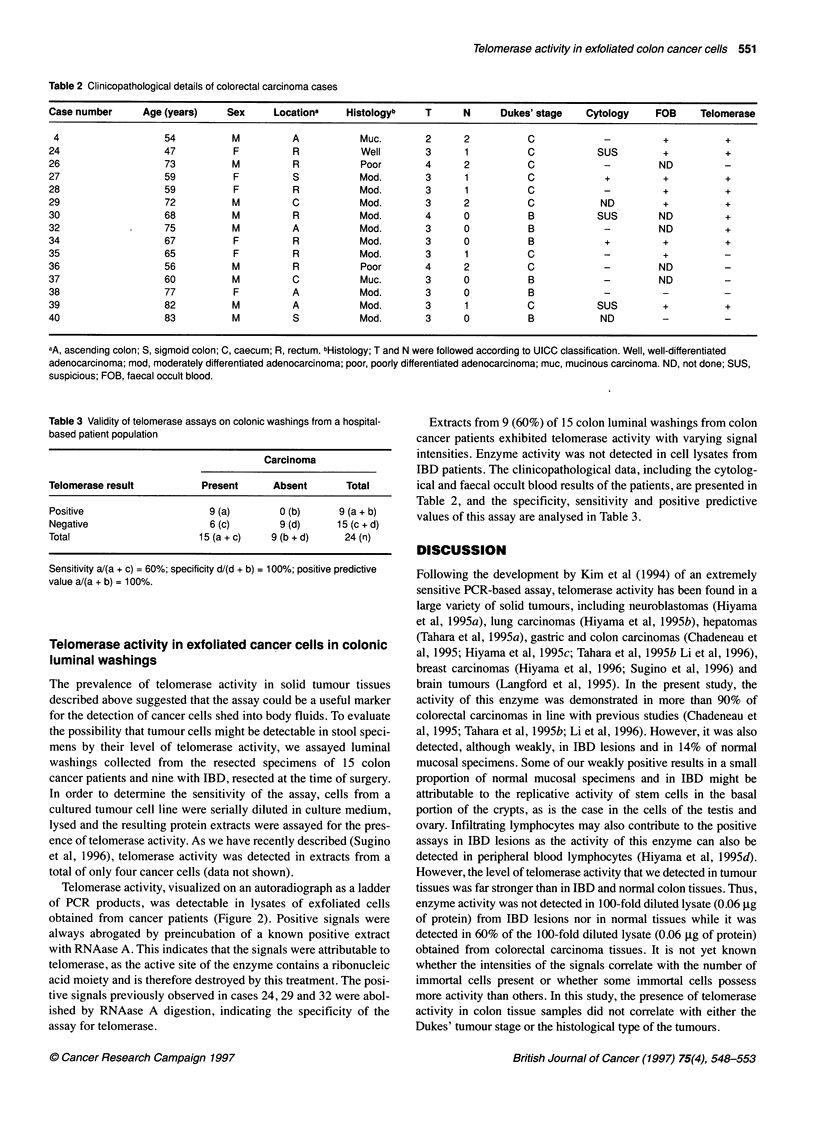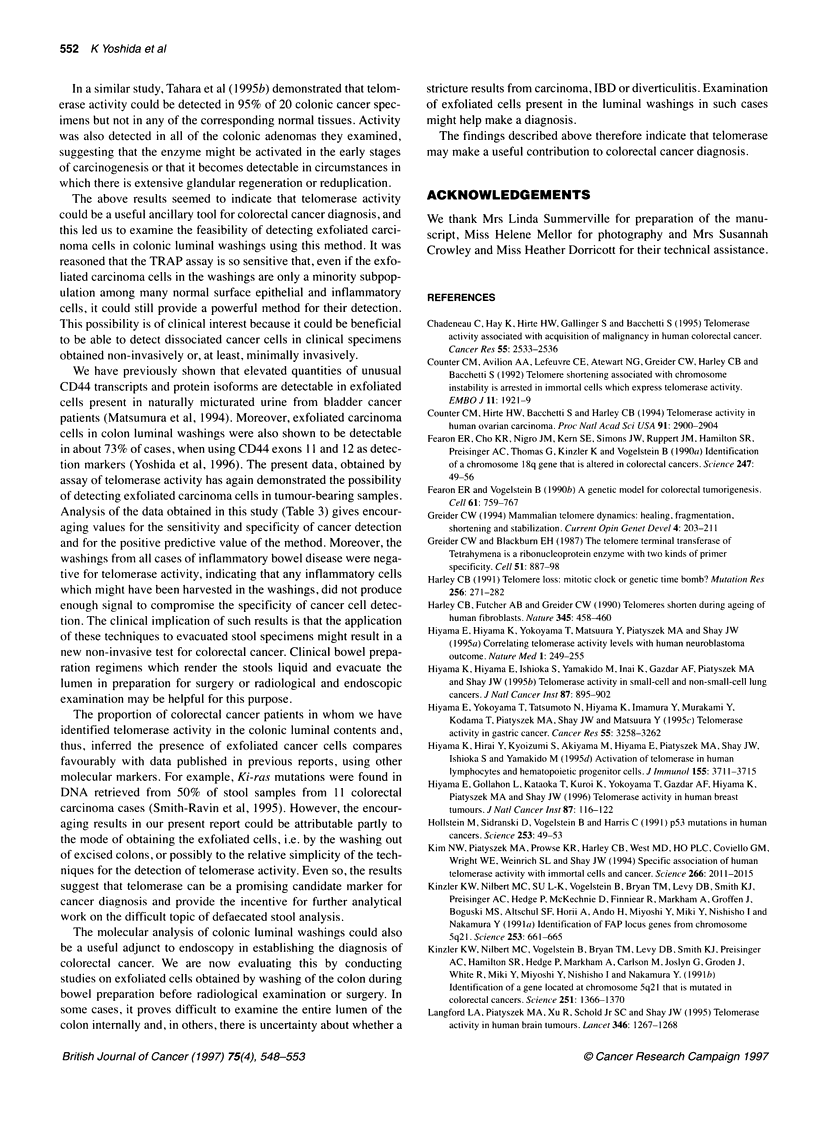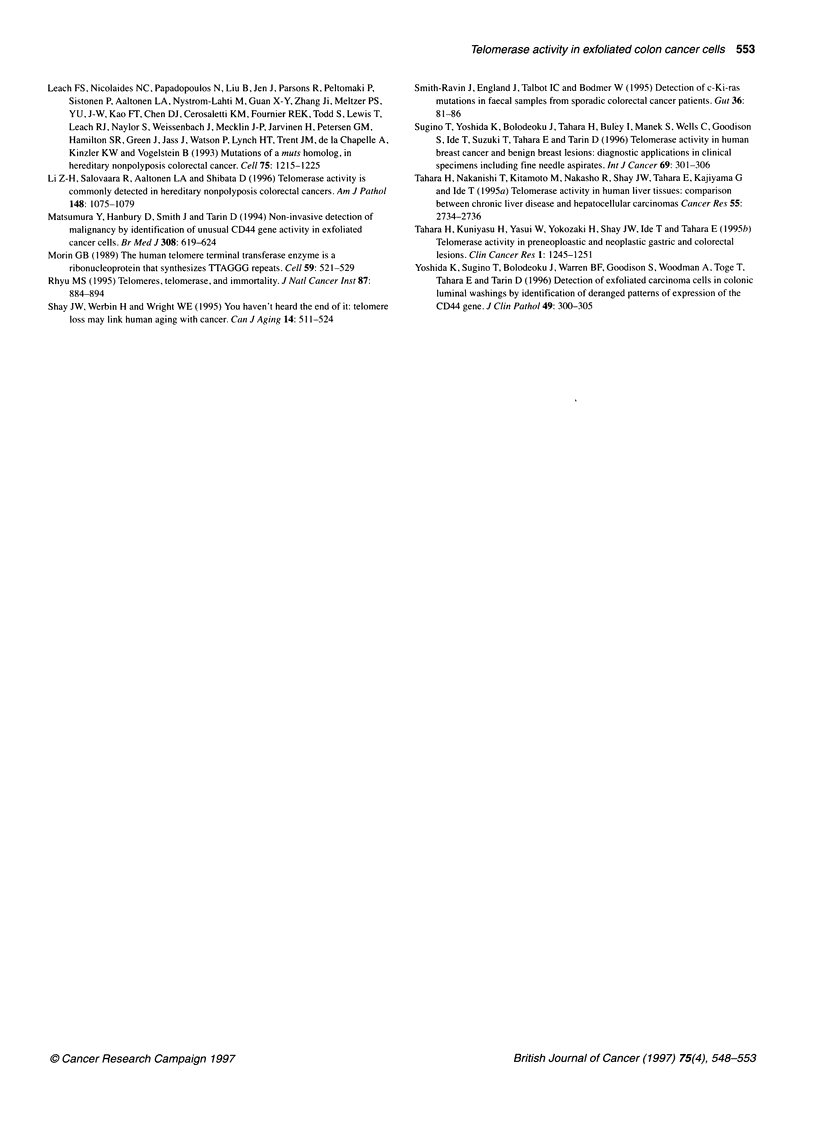# Detection of telomerase activity in exfoliated cancer cells in colonic luminal washings and its related clinical implications.

**DOI:** 10.1038/bjc.1997.96

**Published:** 1997

**Authors:** K. Yoshida, T. Sugino, S. Goodison, B. F. Warren, D. Nolan, S. Wadsworth, N. J. Mortensen, T. Toge, E. Tahara, D. Tarin

**Affiliations:** Nuffield Department of Pathology and Bacteriology, University of Oxford, UK.

## Abstract

**Images:**


British Journal of Cancer (1997) 75(4), 548–553
© 1997 Cancer Research Campaign

### Detection of telomerase activity in exfoliated cancer cells in colonic luminal washings and its related clinical implications

K Yoshida[1], T Sugino[1], S Goodison[1], BF Warren[2], D Nolan[3], S Wadsworth[3], NJ Mortensen[4], T Toge[5], E Tahara[6] and D Tarin[1]

[1]Nuffield Department of Pathology and Bacteriology, University of Oxford; [2]Department of Cellular Pathology, [3]Department of Radiology and [4]Department of Surgery, John Radcliffe Hospital, Headington, Oxford OX3 9DU, UK; [5]Department of Surgical Oncology, Research Institute for Radiation, Biology and Medicine, Hiroshima University; [6]Department of Pathology, School of Medicine Hiroshima University, 1–2–3 Kasumi, Minami-ku, Hiroshima, 734 Japan

**Summary** Telomerase is a ribonucleoprotein capable of replacing telomeric DNA sequences that are lost at each cell division. Under normal circumstances, it is active in rapidly dividing embryonic cells and in stem cell populations but not in terminally differentiated somatic cells. Much attention has recently focused on the hypothesis that activity of this enzyme is necessary for cells to become immortal. This predicts that telomerase activity should be detectable in malignant cells and tissues but not in their normal counterparts, which slowly senesce and die. In accordance with this notion, telomerase activity has been reported in a wide range of malignancies, including those of the gastrointestinal tract, breast and lung. In the present study, we used a polymerase chain reaction (PCR)-based assay for telomerase activity, designated the 'telomeric repeat amplification protocol (TRAP)', to examine initially 35 colonic carcinomas, their corresponding normal tissues and 12 inflammatory bowel disease (IBD) lesions. We detected strong enzyme activity in 32 (92%) of the 35 colon carcinomas while there was no activity in 30 (86%) of 35 matched normal colonic tissue specimens and only very weak activity in the remainder. Four of seven specimens of ulcerative colitis and two of five Crohn's disease lesions were negative, and the rest were only weakly positive. These results led us to examine whether telomerase could be detected in carcinoma cells exfoliated into the colonic lumen. We assayed lysates of exfoliated cells in luminal washings from colectomy specimens of 15 patients with colon carcinoma and nine with IBD. Telomerase activity was detected in washings from 9 (60%) of the 15 colon carcinoma cases but not in any from cases with IBD, suggesting that it can be a good marker for the detection of colon carcinoma, possibly even in non-invasively obtained samples.

**Keywords:** colon carcinoma; exfoliated cancer cells; telomerase activity; non-invasive detection of cancer

Colorectal carcinoma is a common gastrointestinal neoplasm in many countries. Recent advances in surgical treatment, chemotherapy, radiotherapy and immunotherapy have greatly improved the prognosis of such patients. However, despite improvements in the clinical investigation of bowel disorders, including double-contrast barium enema and endoscopy, the early detection of colorectal carcinoma is still a major clinical problem. These methods are expensive, uncomfortable and labour intensive. Conversely, the presence of fresh or occult blood in the stool is a frequent feature in symptomatic disease and is an easy test for screening but it is not specific for carcinoma. A reliable, preferably non-invasive, method is therefore urgently needed for evaluation of symptomatic and asymptomatic individuals, especially high-risk patients.

One approach to this problem is to identify reliable molecular biomarkers for the detection of colon carcinomas, and a number of candidates have recently been suggested. These include mutations in tumour-suppressor genes such as *p53* (Hollstein et al, 1991), *APC* (Kinzler et al, 1991*a*), *MCC* (Kinzler et al, 1991*b*) and *DCC* (Fearon et al, 1990*a*). Mutations in DNA mismatch repair genes (Leach et al, 1993) and oncogenes such as *Ki-ras* (Fearon et al, 1990*b*; Smith-Ravin et al, 1995) have also been implicated.

*Received 23 May 1996*
*Revised 4 September 1996*
*Accepted 13 September 1996*

*Correspondence to*: D Tarin

More recently, the enzyme telomerase has been attracting interest as another promising candidate marker (Counter et al, 1994). The 'telomere hypothesis' proposes that activation of telomerase is necessary for cells to become immortal or capable of extended proliferation (Rhyu, 1995; Shay et al, 1995). Telomeres are specialized nucleoprotein structures at the ends of eukaryotic chromosomes which contain multiple tandem repeats (Greider et al, 1987; Morin, 1989). They are believed to have a role in protecting the end of the chromosome from fusion and recombination events by interactions with DNA-binding proteins and with those of the nuclear matrix. Somatic cell telomeres are progressively shortened by 40–200 bp with each cell division, and reduction beyond a critical point leads to subsequent exit from the cell cycle and senescence (Harley et al, 1990, 1991; Counter et al, 1992; Greider, 1994). The restoration of telomeric repeats to the ends of the chromosome may overcome this limitation, and this maintenance function is performed by the enzyme telomerase.

Telomerase is a ribonucleoprotein that synthesizes TTAGGG tandem repeats at each telomeric region to re-extend it to its original length (Morin, 1989). The enzyme is active in embryonic cells and in stem cells but activity is undetectable in normal, terminally differentiated somatic cells (Kim et al, 1994). Cells that can overcome this limitation have the potential for prolonged survival and indefinite proliferation, and recent data indicate that telomerase is indeed reactivated in immortal cancer cells. Activation of telomerase in a cell may lead to the evolution of a clonal line with enhanced survival capabilities and the potential to develop into a tumour (Harley, 1991; Rhyu, 1995; Shay et al, 1995). Hence, if the 'telomere hypothesis' applies to human malignancy, one might predict that telomerase activity would be detectable in the initial stages of neoplasia and could therefore be a particularly useful marker for early diagnosis.

In the present study, we assessed whether telomerase activity could be a good diagnostic marker of colonic carcinoma. The enzyme activity was initially assayed in colorectal carcinoma tissues, matched adjacent normal tissues and IBD lesions. Furthermore, to elucidate whether it would be possible to detect cancer cells in colonic luminal washings, telomerase activity was assayed in the lysate of exfoliated cells obtained from colonic luminal washings of resected specimens. This study demonstrates the feasibility of non-invasive colonic cancer detection by analysis of colonic luminal washings or stool samples from patients.

### MATERIALS AND METHODS

### Procurement of tissues and exfoliated cells from colonic washings

Surgically resected tissue samples from 35 colorectal carcinomas, their corresponding normal counterparts and 12 IBD lesions, including seven specimens of ulcerative colitis and five of Crohn's disease, were snap frozen and stored in liquid nitrogen until use. The presence of viable carcinoma cells in tissue obtained from cancer specimens and the suitability of normal tissues for use as controls was routinely confirmed by cryostat sectioning before analysis.

Exfoliated cells were collected from 15 colorectal cancer specimens and from nine affected by IBD. The IBD group included five ulcerative colitis and four Crohn's disease specimens. The lumen of each surgically resected specimen was washed with water to remove faecal debris and then with 500 ml of physiological saline, which was collected before opening the bowel for pathological examination and tissue sampling. A 1-ml aliquot of the saline washings was removed for cytology after which the remainder was centrifuged at 3000 r.p.m. for 15 min. The resulting cell pellets were snap frozen in liquid nitrogen and stored at –80°C.

### Protein extraction

Protein extractions from cell lines were performed according to protocols published previously (Kim et al, 1994). The HT29 human colon carcinoma cell line was routinely cultured in RPMI 1640 medium (Gibco, BRL, Paisley, UK) supplemented with 10% fetal calf serum at 37°C in an atmosphere containing 5% carbon dioxide. Cells were harvested and centrifuged at 3000 r.p.m. for 5 min and the cell pellet was washed with ice-cold washing buffer [10 mM Hepes–potassium hydroxide (pH 7.5), 1.5 mM magnesium chloride, 10 mM potassium chloride, 1 mM dithiothreitol], pelleted again and resuspended in four volumes of the cell pellet of ice-cold TRAP lysis buffer [10 mM Tris-HCl (pH 7.5), 1 mM magnesium chloride, 1 mM EGTA, 0.1 mM AEBSF (ICN Biomedicals, Thame, UK), 5 mM β-mercaptoethanol, 0.5% CHAPS, 10% glycerol].

**Figure 1** Telomerase activity in colon carcinoma tissues and in normal colonic tissues (cases 71, 72, 73, 76). T represents tumour tissues and N represents normal tissues. Six micrograms of tissue lysate protein was used for each reaction. Positive results consisting of intense extended 6 bp ladders were clearly observed in tumour tissues. Normal tissues showed either no signal or occasionally a faint short ladder (e.g. case 72). The enzyme activity in the cell lysate (6 μg of protein) was also examined in ulcerative colitis (UC) and Crohn's disease (CD) lesions. Weak enzyme activity was observed in the lysate of UC patient cases 2 and 3 and CD patient cases 1 and 2. Cell lysate (6 μg of protein) from a HT29 human colon carcinoma cell line was used as a positive control. The activity was not detected in a reaction mixture containing no cell lysate

© Cancer Research Campaign 1997

Tissues from colorectal carcinomas and from matched normal mucosa were histologically verified by cryostat sectioning before analysis. For protein extraction, 20 cryostat sections (10-μm thick) from each sample, were dissolved in 50–70 μl of ice-cold TRAP lysis buffer, incubated for 30 min on ice and centrifuged for 30 min at 14 000 r.p.m. at 4°C. The supernatant was decanted, snap frozen and stored at –80°C. The frozen pellet of exfoliated cells from colon luminal washings were washed with washing buffer and then dissolved in the lysis buffer of four cell-pellet volumes and lysed in the same manner as tissues. Protein concentrations were measured by Bio-Rad protein assay kit (Bio-Rad, UK) and were in the range of 5–10 mg $ml^{-1}$. Six micrograms of the extract were used for each telomerase assay.

### Telomerase assay

Telomerase activity was assayed by a modification of the 'telomeric repeat amplification protocol (TRAP)' (Kim et al, 1994). Briefly, 2 μl of the cell extract (3 μg protein $\mu l^{-1}$) were incubated with 20 mM Tris-HCl (pH 8.3), 1 mM magnesium chloride, 63 mM potassium chloride, 0.005% Tween-20, 1 mM EGTA, 50 mM deoxynucleotide triphosphate, 0.4 μl of [$\alpha$-$^{32}$P]dCTP (10 mCi $ml^{-1}$, 3000 Ci $mmol^{-1}$, Amersham, UK), 1 μg of T4g32 protein (Boehringer Mannheim), bovine serum albumin (0.1 mg $ml^{-1}$), 2 units of *Taq* DNA polymerase (Boehringer Mannheim) and 0.1 μg of TS primer (5′-AATCCGTCGAGCAGAGTT-3′) at 20°C for 30 min and then heated at 90°C for 3 min. During the latter step, 0.1 μg of CX primer (5′-CCCTTACCCTTACCCTTACCCT AA-3′) was added, and the reaction mixture was subjected to 31 PCR cycles of 94°C for 45 s, 50°C for 45 s and 72°C for 90 s. For assessment of the sensitivity of the telomerase assay, extracts containing 6 μg, 0.6 μg and 0.06 μg of protein were routinely used in the TRAP reaction. Confirmation of the RNAase A-sensitivity of positive telomerase assays was performed as follows: 10 μl of cell extract were digested with 0.5 μg of RNAase A (Boehringer) for 20 min at 37°C, and 2 μl of the protein extract was used in the TRAP reaction. The TRAP assay results were visualized by electrophoresis of 15 μl of the PCR products in 0.5 × Tris-borate EDTA buffer in 12% polyacrylamide non-denaturing gels. Gels were dried and exposed to radiographic film overnight at room temperature.

### RESULTS

### Telomerase activity in colorectal carcinoma tissues

In this assay, telomerase activity is recognized by a distinctive series of bands present in an electrophoretic gel or on autoradiographic film. Each band differs in size from its neighbour by the addition of a further TTAGGG repeat, creating the appearance of a 6 bp ladder (Figure 1).

Telomerase activity was detected in 32 (92%) of the 35 colorectal carcinomas examined, while it was not detected in 30 (86%) of the 35 matched normal tissues from the same patients and was only very weakly present in the remainder. Representative results are shown in Figure 1. In this figure, it can be seen that, although telomerase activity was detected in all the tumour samples shown, the intensity of the signal differed from case to case. An example of a weakly positive normal tissue sample is also shown (case 72). In order to compare the intensity of the activity present in the different samples, cell lysates were serially diluted before the TRAP assay was performed. Telomerase activity could not be detected in normal tissue lysates which were diluted 100-fold (0.06 μg). However, it was detected in 60% of colon carcinoma tissue lysates, even after 100-fold dilution (0.06 μg). The clinicopathological details of each of the carcinoma cases are compared with the corresponding observed activity of the enzyme in Table 1. There was no correlation between telomerase activity and tumour stage. The enzyme activity was also measured in IBD lesions (Figure 1). Although it was very much weaker in these lesions than in carcinomas, activity was detected in three of seven specimens of ulcerative colitis and three of five specimens of Crohn's disease.

**Table 1** Telomerase activity of colon carcinoma tissues

| | Telomerase activity | |
|---|---|---|
| | **Undetectable (*n* = 3)** | **Detectable (*n* = 32)** |
| Age at diagnosis (year) | 66–76 | 47–85 |
| Mean age at diagnosis | 72 | 68 |
| Sex (male–female) | 0 : 3 | 21 : 11 |
| Tumour stage (Dukes) | | |
| A | 0 | 4 |
| B | 0 | 16 |
| C | 2 | 12 |
| D | 1 | 0 |
| Histology[a] | | |
| Well | 0 | 4 |
| Mod. | 2 | 24 |
| Poor, Muc., Signet | 1 | 4 |
| | | [32/35 (92%)] |

[a]Well, well-differentiated adenocarcinoma; Mod, moderately differentiated adenocarcinoma; Poor, poorly differentiated adenocarcinoma; Muc, mucinous carcinoma; Signet, signet ring cell carcinoma.

**Figure 2** Telomerase activity in exfoliated cell lysates from colon luminal washings of cancer patients (cases 24, 29 and 32). Telomerase activity was detected in cell lysates (6 μg of protein) from cancer patients, and its activity was not detected in the RNAase A-pretreated cell lysates

*© Cancer Research Campaign 1997*

**Table 2** Clinicopathological details of colorectal carcinoma cases

| Case number | Age (years) | Sex | Location[a] | Histology[b] | T | N | Dukes' stage | Cytology | FOB | Telomerase |
|---|---|---|---|---|---|---|---|---|---|---|
| 4 | 54 | M | A | Muc. | 2 | 2 | C | – | + | + |
| 24 | 47 | F | R | Well | 3 | 1 | C | SUS | + | + |
| 26 | 73 | M | R | Poor | 4 | 2 | C | – | ND | – |
| 27 | 59 | F | S | Mod. | 3 | 1 | C | + | + | + |
| 28 | 59 | F | R | Mod. | 3 | 1 | C | – | + | + |
| 29 | 72 | M | C | Mod. | 3 | 2 | C | ND | + | + |
| 30 | 68 | M | R | Mod. | 4 | 0 | B | SUS | ND | + |
| 32 | 75 | M | A | Mod. | 3 | 0 | B | – | ND | + |
| 34 | 67 | F | R | Mod. | 3 | 0 | B | + | + | + |
| 35 | 65 | F | R | Mod. | 3 | 1 | C | – | + | – |
| 36 | 56 | M | R | Poor | 4 | 2 | C | – | ND | – |
| 37 | 60 | M | C | Muc. | 3 | 0 | B | – | ND | – |
| 38 | 77 | F | A | Mod. | 3 | 0 | B | – | – | – |
| 39 | 82 | M | A | Mod. | 3 | 1 | C | SUS | + | + |
| 40 | 83 | M | S | Mod. | 3 | 0 | B | ND | – | – |

[a]A, ascending colon; S, sigmoid colon; C, caecum; R, rectum. [b]Histology; T and N were followed according to UICC classification. Well, well-differentiated adenocarcinoma; mod, moderately differentiated adenocarcinoma; poor, poorly differentiated adenocarcinoma; muc, mucinous carcinoma. ND, not done; SUS, suspicious; FOB, faecal occult blood.

**Table 3** Validity of telomerase assays on colonic washings from a hospital-based patient population

| Telomerase result | Carcinoma Present | Carcinoma Absent | Total |
|---|---|---|---|
| Positive | 9 (a) | 0 (b) | 9 (a + b) |
| Negative | 6 (c) | 9 (d) | 15 (c + d) |
| Total | 15 (a + c) | 9 (b + d) | 24 (n) |

Sensitivity a/(a + c) = 60%; specificity d/(d + b) = 100%; positive predictive value a/(a + b) = 100%.

### Telomerase activity in exfoliated cancer cells in colonic luminal washings

The prevalence of telomerase activity in solid tumour tissues described above suggested that the assay could be a useful marker for the detection of cancer cells shed into body fluids. To evaluate the possibility that tumour cells might be detectable in stool specimens by their level of telomerase activity, we assayed luminal washings collected from the resected specimens of 15 colon cancer patients and nine with IBD, resected at the time of surgery. In order to determine the sensitivity of the assay, cells from a cultured tumour cell line were serially diluted in culture medium, lysed and the resulting protein extracts were assayed for the presence of telomerase activity. As we have recently described (Sugino et al, 1996), telomerase activity was detected in extracts from a total of only four cancer cells (data not shown).

Telomerase activity, visualized on an autoradiograph as a ladder of PCR products, was detectable in lysates of exfoliated cells obtained from cancer patients (Figure 2). Positive signals were always abrogated by preincubation of a known positive extract with RNAase A. This indicates that the signals were attributable to telomerase, as the active site of the enzyme contains a ribonucleic acid moiety and is therefore destroyed by this treatment. The positive signals previously observed in cases 24, 29 and 32 were abolished by RNAase A digestion, indicating the specificity of the assay for telomerase.

Extracts from 9 (60%) of 15 colon luminal washings from colon cancer patients exhibited telomerase activity with varying signal intensities. Enzyme activity was not detected in cell lysates from IBD patients. The clinicopathological data, including the cytological and faecal occult blood results of the patients, are presented in Table 2, and the specificity, sensitivity and positive predictive values of this assay are analysed in Table 3.

### DISCUSSION

Following the development by Kim et al (1994) of an extremely sensitive PCR-based assay, telomerase activity has been found in a large variety of solid tumours, including neuroblastomas (Hiyama et al, 1995*a*), lung carcinomas (Hiyama et al, 1995*b*), hepatomas (Tahara et al, 1995*a*), gastric and colon carcinomas (Chadeneau et al, 1995; Hiyama et al, 1995*c*; Tahara et al, 1995*b* Li et al, 1996), breast carcinomas (Hiyama et al, 1996; Sugino et al, 1996) and brain tumours (Langford et al, 1995). In the present study, the activity of this enzyme was demonstrated in more than 90% of colorectal carcinomas in line with previous studies (Chadeneau et al, 1995; Tahara et al, 1995*b*; Li et al, 1996). However, it was also detected, although weakly, in IBD lesions and in 14% of normal mucosal specimens. Some of our weakly positive results in a small proportion of normal mucosal specimens and in IBD might be attributable to the replicative activity of stem cells in the basal portion of the crypts, as is the case in the cells of the testis and ovary. Infiltrating lymphocytes may also contribute to the positive assays in IBD lesions as the activity of this enzyme can also be detected in peripheral blood lymphocytes (Hiyama et al, 1995*d*). However, the level of telomerase activity that we detected in tumour tissues was far stronger than in IBD and normal colon tissues. Thus, enzyme activity was not detected in 100-fold diluted lysate (0.06 μg of protein) from IBD lesions nor in normal tissues while it was detected in 60% of the 100-fold diluted lysate (0.06 μg of protein) obtained from colorectal carcinoma tissues. It is not yet known whether the intensities of the signals correlate with the number of immortal cells present or whether some immortal cells possess more activity than others. In this study, the presence of telomerase activity in colon tissue samples did not correlate with either the Dukes' tumour stage or the histological type of the tumours.

© *Cancer Research Campaign 1997*

In a similar study, Tahara et al (1995*b*) demonstrated that telomerase activity could be detected in 95% of 20 colonic cancer specimens but not in any of the corresponding normal tissues. Activity was also detected in all of the colonic adenomas they examined, suggesting that the enzyme might be activated in the early stages of carcinogenesis or that it becomes detectable in circumstances in which there is extensive glandular regeneration or reduplication.

The above results seemed to indicate that telomerase activity could be a useful ancillary tool for colorectal cancer diagnosis, and this led us to examine the feasibility of detecting exfoliated carcinoma cells in colonic luminal washings using this method. It was reasoned that the TRAP assay is so sensitive that, even if the exfoliated carcinoma cells in the washings are only a minority subpopulation among many normal surface epithelial and inflammatory cells, it could still provide a powerful method for their detection. This possibility is of clinical interest because it could be beneficial to be able to detect dissociated cancer cells in clinical specimens obtained non-invasively or, at least, minimally invasively.

We have previously shown that elevated quantities of unusual CD44 transcripts and protein isoforms are detectable in exfoliated cells present in naturally micturated urine from bladder cancer patients (Matsumura et al, 1994). Moreover, exfoliated carcinoma cells in colon luminal washings were also shown to be detectable in about 73% of cases, when using CD44 exons 11 and 12 as detection markers (Yoshida et al, 1996). The present data, obtained by assay of telomerase activity has again demonstrated the possibility of detecting exfoliated carcinoma cells in tumour-bearing samples. Analysis of the data obtained in this study (Table 3) gives encouraging values for the sensitivity and specificity of cancer detection and for the positive predictive value of the method. Moreover, the washings from all cases of inflammatory bowel disease were negative for telomerase activity, indicating that any inflammatory cells which might have been harvested in the washings, did not produce enough signal to compromise the specificity of cancer cell detection. The clinical implication of such results is that the application of these techniques to evacuated stool specimens might result in a new non-invasive test for colorectal cancer. Clinical bowel preparation regimens which render the stools liquid and evacuate the lumen in preparation for surgery or radiological and endoscopic examination may be helpful for this purpose.

The proportion of colorectal cancer patients in whom we have identified telomerase activity in the colonic luminal contents and, thus, inferred the presence of exfoliated cancer cells compares favourably with data published in previous reports, using other molecular markers. For example, *Ki-ras* mutations were found in DNA retrieved from 50% of stool samples from 11 colorectal carcinoma cases (Smith-Ravin et al, 1995). However, the encouraging results in our present report could be attributable partly to the mode of obtaining the exfoliated cells, i.e. by the washing out of excised colons, or possibly to the relative simplicity of the techniques for the detection of telomerase activity. Even so, the results suggest that telomerase can be a promising candidate marker for cancer diagnosis and provide the incentive for further analytical work on the difficult topic of defaecated stool analysis.

The molecular analysis of colonic luminal washings could also be a useful adjunct to endoscopy in establishing the diagnosis of colorectal cancer. We are now evaluating this by conducting studies on exfoliated cells obtained by washing of the colon during bowel preparation before radiological examination or surgery. In some cases, it proves difficult to examine the entire lumen of the colon internally and, in others, there is uncertainty about whether a stricture results from carcinoma, IBD or diverticulitis. Examination of exfoliated cells present in the luminal washings in such cases might help make a diagnosis.

The findings described above therefore indicate that telomerase may make a useful contribution to colorectal cancer diagnosis.

### ACKNOWLEDGEMENTS

We thank Mrs Linda Summerville for preparation of the manuscript, Miss Helene Mellor for photography and Mrs Susannah Crowley and Miss Heather Dorricott for their technical assistance.

### REFERENCES

Chadeneau C, Hay K, Hirte HW, Gallinger S and Bacchetti S (1995) Telomerase activity associated with acquisition of malignancy in human colorectal cancer. *Cancer Res* **55**: 2533–2536

Counter CM, Avilion AA, Lefeuvre CE, Atewart NG, Greider CW, Harley CB and Bacchetti S (1992) Telomere shortening associated with chromosome instability is arrested in immortal cells which express telomerase activity. *EMBO J* **11**: 1921–9

Counter CM, Hirte HW, Bacchetti S and Harley CB (1994) Telomerase activity in human ovarian carcinoma. *Proc Natl Acad Sci USA* **91**: 2900–2904

Fearon ER, Cho KR, Nigro JM, Kern SE, Simons JW, Ruppert JM, Hamilton SR, Preisinger AC, Thomas G, Kinzler K and Vogelstein B (1990*a*) Identification of a chromosome 18q gene that is altered in colorectal cancers. *Science* **247**: 49–56

Fearon ER and Vogelstein B (1990*b*) A genetic model for colorectal tumorigenesis. *Cell* **61**: 759–767

Greider CW (1994) Mammalian telomere dynamics: healing, fragmentation, shortening and stabilization. *Current Opin Genet Devel* **4**: 203–211

Greider CW and Blackburn EH (1987) The telomere terminal transferase of Tetrahymena is a ribonucleoprotein enzyme with two kinds of primer specificity. *Cell* **51**: 887–98

Harley CB (1991) Telomere loss: mitotic clock or genetic time bomb? *Mutation Res* **256**: 271–282

Harley CB, Futcher AB and Greider CW (1990) Telomeres shorten during ageing of human fibroblasts. *Nature* **345**: 458–460

Hiyama E, Hiyama K, Yokoyama T, Matsuura Y, Piatyszek MA and Shay JW (1995*a*) Correlating telomerase activity levels with human neuroblastoma outcome. *Nature Med* **1**: 249–255

Hiyama K, Hiyama E, Ishioka S, Yamakido M, Inai K, Gazdar AF, Piatyszek MA and Shay JW (1995*b*) Telomerase activity in small-cell and non-small-cell lung cancers. *J Natl Cancer Inst* **87**: 895–902

Hiyama E, Yokoyama T, Tatsumoto N, Hiyama K, Imamura Y, Murakami Y, Kodama T, Piatyszek MA, Shay JW and Matsuura Y (1995*c*) Telomerase activity in gastric cancer. *Cancer Res* **55**: 3258–3262

Hiyama K, Hirai Y, Kyoizumi S, Akiyama M, Hiyama E, Piatyszek MA, Shay JW, Ishioka S and Yamakido M (1995*d*) Activation of telomerase in human lymphocytes and hematopoietic progenitor cells. *J Immunol* **155**: 3711–3715

Hiyama E, Gollahon L, Kataoka T, Kuroi K, Yokoyama T, Gazdar AF, Hiyama K, Piatyszek MA and Shay JW (1996) Telomerase activity in human breast tumours. *J Natl Cancer Inst* **87**: 116–122

Hollstein M, Sidranski D, Vogelstein B and Harris C (1991) p53 mutations in human cancers. *Science* **253**: 49–53

Kim NW, Piatyszek MA, Prowse KR, Harley CB, West MD, HO PLC, Coviello GM, Wright WE, Weinrich SL and Shay JW (1994) Specific association of human telomerase activity with immortal cells and cancer. *Science* **266**: 2011–2015

Kinzler KW, Nilbert MC, SU L-K, Vogelstein B, Bryan TM, Levy DB, Smith KJ, Preisinger AC, Hedge P, McKechnie D, Finniear R, Markham A, Groffen J, Boguski MS, Altschul SF, Horii A, Ando H, Miyoshi Y, Miki Y, Nishisho I and Nakamura Y (1991*a*) Identification of FAP locus genes from chromosome 5q21. *Science* **253**: 661–665

Kinzler KW, Nilbert MC, Vogelstein B, Bryan TM, Levy DB, Smith KJ, Preisinger AC, Hamilton SR, Hedge P, Markham A, Carlson M, Joslyn G, Groden J, White R, Miki Y, Miyoshi Y, Nishisho I and Nakamura Y. (1991*b*) Identification of a gene located at chromosome 5q21 that is mutated in colorectal cancers. *Science* **251**: 1366–1370

Langford LA, Piatyszek MA, Xu R, Schold Jr SC and Shay JW (1995) Telomerase activity in human brain tumours. *Lancet* **346**: 1267–1268

© Cancer Research Campaign 1997

Leach FS, Nicolaides NC, Papadopoulos N, Liu B, Jen J, Parsons R, Peltomaki P, Sistonen P, Aaltonen LA, Nystrom-Lahti M, Guan X-Y, Zhang Ji, Meltzer PS, YU, J-W, Kao FT, Chen DJ, Cerosaletti KM, Fournier REK, Todd S, Lewis T, Leach RJ, Naylor S, Weissenbach J, Mecklin J-P, Jarvinen H, Petersen GM, Hamilton SR, Green J, Jass J, Watson P, Lynch HT, Trent JM, de la Chapelle A, Kinzler KW and Vogelstein B (1993) Mutations of a *muts* homolog, in hereditary nonpolyposis colorectal cancer. *Cell* **75**: 1215–1225

Li Z-H, Salovaara R, Aaltonen LA and Shibata D (1996) Telomerase activity is commonly detected in hereditary nonpolyposis colorectal cancers. *Am J Pathol* **148**: 1075–1079

Matsumura Y, Hanbury D, Smith J and Tarin D (1994) Non-invasive detection of malignancy by identification of unusual CD44 gene activity in exfoliated cancer cells. *Br Med J* **308**: 619–624

Morin GB (1989) The human telomere terminal transferase enzyme is a ribonucleoprotein that synthesizes TTAGGG repeats. *Cell* **59**: 521–529

Rhyu MS (1995) Telomeres, telomerase, and immortality. *J Natl Cancer Inst* **87**: 884–894

Shay JW, Werbin H and Wright WE (1995) You haven't heard the end of it: telomere loss may link human aging with cancer. *Can J Aging* **14**: 511–524

Smith-Ravin J, England J, Talbot IC and Bodmer W (1995) Detection of c-Ki-ras mutations in faecal samples from sporadic colorectal cancer patients. *Gut* **36**: 81–86

Sugino T, Yoshida K, Bolodeoku J, Tahara H, Buley I, Manek S, Wells C, Goodison S, Ide T, Suzuki T, Tahara E and Tarin D (1996) Telomerase activity in human breast cancer and benign breast lesions: diagnostic applications in clinical specimens including fine needle aspirates. *Int J Cancer* **69**: 301–306

Tahara H, Nakanishi T, Kitamoto M, Nakasho R, Shay JW, Tahara E, Kajiyama G and Ide T (1995*a*) Telomerase activity in human liver tissues: comparison between chronic liver disease and hepatocellular carcinomas *Cancer Res* **55**: 2734–2736

Tahara H, Kuniyasu H, Yasui W, Yokozaki H, Shay JW, Ide T and Tahara E (1995*b*) Telomerase activity in preneoploastic and neoplastic gastric and colorectal lesions. *Clin Cancer Res* **1**: 1245–1251

Yoshida K, Sugino T, Bolodeoku J, Warren BF, Goodison S, Woodman A, Toge T, Tahara E and Tarin D (1996) Detection of exfoliated carcinoma cells in colonic luminal washings by identification of deranged patterns of expression of the CD44 gene. *J Clin Pathol* **49**: 300–305

© Cancer Research Campaign 1997